# Prevalence and associated factors of HIV testing among young (15–24) women in eastern Africa: a multilevel analysis of demographic health survey data (2008-2018)

**DOI:** 10.1186/s13690-022-00879-2

**Published:** 2022-04-11

**Authors:** Misganaw Gebrie Worku, Achamyeleh Birhanu Teshale, Getayeneh Antehunegn Tesema

**Affiliations:** 1 Department of Human Anatomy, University of Gondar, college of medicine and health science, School of Medicine, Gondar, Ethiopia; 2 Department of Epidemiology and Biostatistics, Institute of Public Health, College of Medicine and Health Sciences, University of Gondar, Gondar, Ethiopia

**Keywords:** HIV/AIDS, Multilevel analysis, East Africa, Young women

## Abstract

**Background:**

According to available evidence, only 15% of young women in sub-Saharan Africa know their Human immune deficiency virus (HIV) status. Despite a high prevalence of HIV infection among adolescents and young women, policymakers give less attention to HIV testing and counseling services. So, this study aimed to investigate the pooled prevalence and associated factors of HIV testing among young women in east Africa.

**Methods:**

The most recent DHS surveys done among 11 east African countries were pooled and a weighted sample of 73,661 young women were included. At bivariable analysis variables with a *p*-value≤0.2 were selected for multivariable analysis and variables with a *p*-value of ≤0.05 in the multivariable analysis were considered as a statistically significant determinant of HIV testing.

**Results:**

Pooled prevalence of HIV testing among young women was 55.3%: 95% CI (54.97%, 55.69%). In the multilevel multivariable analysis: respondent age, marital status, educational level, occupation, media exposure, having higher and comprehensive knowledge about HIV / AIDS, having some and higher risky sexual behavior, visiting health care facilities, being rural dweller, being from rich households, having multiple sexual partners, early sex initiation and community-level education were significantly associated with HIV testing.

**Conclusion:**

The prevalence of HIV testing among young women was significantly affected by both individual and community-level factors. To prevent the transmission and dissemination of HIV, there should be a systematic and coordinated approach and policy for HIV testing among young people.

## Background

Globally, 36.7 million people were living with human immune deficiency virus (HIV) / Acquired immune deficiency syndrome (AIDS) in 2015 with 70 % of infected people were living in sub-Saharan Africa [1, 2]. Developing countries account for 97% of worldwide HIV cases and Sub-Saharan Africa is the world's first disease-affected region [3]. In 2017, the United Nations Joint AIDS Program reported that approximately 3.9 million young people worldwide were infected with HIV [4]. Around 2.9 million of the 4.9 million young people living with HIV / AIDS live in eastern and southern Africa [5]. Only 15 % of young women aged 15-24 years in sub-Saharan Africa were aware of their HIV status in 2013 [6]. The prevalence of HIV testing among young woman in Africa ranges from 23.5% to 60.10% [4–7]. Despite a high prevalence of HIV infection among adolescents and young women in sub-Saharan Africa, policymakers give less attention to HIV testing and counseling services. Young women are exposed to HIV in two stages of their lives, from mother to child, at an early age and during their adolescence because of their sexual preference and gender differences [8]. Also, young women are vulnerable to HIV due to extreme peer pressure and the emergence of their sexual and social identities [9]. Factors associated with young people's HIV testing include demographic factors (age, education, wealth) [10, 11], HIV risk behavior [12] psychosocial variables associated with HIV awareness [8], and stigma attitudes [12]. Multiple sexual partners [12, 13] and age at sexual initiation [8] were also other factors associated with HIV testing and counseling. Although HIV counseling and testing (HCT) is very important for all strategies related to care, prevention, and treatment of HIV, health care planners and policymakers give less attention, particularly in developing countries [1, 4]. Understanding the factors affecting HIV testing and counseling utilization also helps policymakers to formulate effective strategies for HIV prevention and control. Besides, there is limited studies in HIV testing prevalence and associated factors in the study area using advanced model. Most previous studies in different regions of Africa on the prevalence of HIV testing have been focused on samples of adults and pregnant women [8]. This study extends previous studies focused on the populations of adult and/or pregnant women and attempts to fill the literature gap by addressing the determinants of HIV testing among young women. This study was therefore aimed at investigating the pooled prevalence and factors related to HIV testing among young women in east Africa.

## Methods

### Data sources

A secondary data analysis using the pooled data from recent demographic and health surveys (DHS) conducted in east Africa was done. The data were extracted from the DHS calculation software. The survey of each country consists of men, women, infants, birth and household datasets. The women's datasets (IR files) were used for this analysis. The study included only youth, described by the United nation (UN) as “individuals between the ages of 15 and 24” [14].

The DHS used two stages stratified sampling technique to select the study participants. The women's data of the most recent DHS done in the 11 east African countries were appended. A total weighted sample of 73,661 young women was used for this study. The number of young women included in each country is presented in Table 1.

**Table 1:** Study participants included in the study by country and year of survey in east Africa

Country	Year of survey	Frequency (%)
Burundi	2016	7103 (9.64%)
Ethiopia	2016	6143 (8.33%)
Kenya	2014	11555 (15.68%)
Comoros	2012	2311 (3.13%)
Madagascar	2008	6775 (9.19%)
Malawi	2015/16	10421 (14.15%)
Mozambique	2011	5515 (7.48%)
Rwanda	2014/15	5225 (7.09%)
Uganda	2016	8086 (10.97%)
Zambia	2018	6631 (9%)
Zimbabwe	2013/2014	3895 (5.28%)
Total	73,661	

### Variables

The outcome variable for this study was “ever tested for HIV”. The individual-level factor includes respondent age, marital status, age at first sex, stigmatized attitude, educational statues, household wealth status, knowledge about HIV/AIDS, risky sexual behavior and health care facility visit, number of lifetime sexual partners, media exposure and occupational status. Factors at the community level were community-level women's education, residence and country. The community women's educational level was created by aggregating the individual-level variables, using the proportion of women in the community who were not educated and classified as high and low based on the median value (Table 2).

**Table 2:** Description and measurement of independent variables

Independent variables and their description/categorization	
Individual-level variables	
Age Group	The current age of the women and re-coded into two categories with values of “0” for 15–19, “1” for 20–24.
Wealth Index	The datasets contained a wealth index that was created using principal components analysis coded as “poorest”, “poorer”, “Middle”, “Richer”, and “Richest in the EDHS data set.” For this study we recoded it in to three categories as “poor” (includes the poorest and the poorer categories), “middle”, and “rich” (includes the richer and the richest categories)
Working status	Re-coded in two categories with a value of “0” for not working, and “1” for working.
Visit health facility	Recoded into two categories with a value of “0” for those who visited the health facility in the last 12 months and “1” for those who did not visit the health facility in the past 12 months.
Media exposure	A composite variable obtained by combining whether a respondent reads newspaper/magazine, listen to the radio, and watch television with a value of “0” if a woman were not exposed to at least one of the three media, and “1” if a woman has access/exposure to at least one of the three media.
Educational status	This is the minimum educational level a woman achieved and re-coded into three groups with a value of “0” for no education, “1” for primary education, and “2” for secondary education and “3” for tertiary and above educational level as higher education.
Marital status	This was the current marital status of women and recoded in two categories with a value of “0” for unmarried (includes those who were never in union, divorced, widowed, and separated), and “1” for “married” (includes those living with a partner and those who are married)
Age at sex	The variable age at sex is coded as “0” for those who have first sex before 20 years and “1” for those who initiate sex at 20 and above years.
Risky sexual behavior	By using a different question from the DHS survey it was coded into three categories “0” for no risky sexual behavior, “1” for some risky sexual behavior and “2” for those with higher risk sexual behavior.
HIV knowledge	It was generated from different HIV-related questions from the DHS guide and coded as “0” for those with low knowledge about HIV, “1” for those with high knowledge about HIV/AIDS and “2” for those having comprehensive HIV knowledge.
Stigma indicator	The variable sigma indicator was generated from individual DHS survey questions about an attitude towards HIV and coded as “0” for no stigmatized attitude, “1” for low stigma attitude, “2” for moderate stigma attitude and “3” for those with high stigmatized attitude towards HIV/AIDS.
Number of sexual partner	The variable number of sexual partner coded “0” for those with one sexual partner and “1” for having more than one sexual partner.
Community level variables	
Community of poverty level	Measured by proportion of households in the poor (combination of poorer and poorest) wealth quintile derived from data on wealth index. Then it was categorized based on national median value as: low (communities in which <50 % of women had poor socioeconomic status) and high (communities in which ≥50% of women had poor socioeconomic status) poverty level.
Type of place of residence	The variable place of residence recorded as rural and urban in the dataset was used without change.

### Operational definition

HIV knowledge: generated based on three questions related to HIV prevention and three questions related to the modes of HIV transmission and graded as low (if a woman answered ≤3 questions), high (if a woman answered 4–5 questions), or comprehensive (if a woman answered all the 6 questions) [15].

Risky sexual behaviors: assessed based on five questions; having had STI in last 12 months, genital sore/ulcer in last 12 months, genital discharge in last 12 months, having at least one sexual partner other than the husband in the last 12 months and multiple lifetimes sexual partnership. These were combined into an index of risky sexual behavior with three categories: “no risk” (if the response is no for all questions), “some risk” (if the response is yes for one of the five questions) and “high risk” (if the response is yes to at least two questions) [15].

HIV Stigma indicator: Six questions, which indicate negative attitudes towards people living with HIV / AIDS were used to this variable. This was categorized as “no stigma” (if we got a score of 6), “low stigma” (if we got a score of 4–5), “moderate stigma” (if we got a score of 2–3), and “High stigma” (if we got only score 1) [15].

### Data management and analysis

The data was extracted, recorded and analyzed using Stata 14 software. For the representativeness of the data and to have an accurate estimate and standard error, weighting was done before any statistical analysis. Since the DHS data violates the independent assumptions of the standard logistic regression model, a multilevel logistic regression was fitted. Also, consideration of variability among clusters was addressed through the use of these advanced models. To determine whether or not there was a clustering, the Interclass Correlation Coefficient (ICC) and Median Odds Ratio (MOR) were calculated. Deviance (-2LL) was used for model comparison. Four models: null model - a model without explanatory variables, model I - a model with individual-level factors, model II - a model with community-level factors and model III - a model with both individual and community-level factors were fitted. Since it had the lowest deviance among all fitted models, model III was selected. Multi-level logistic regression was performed with both bivariable and multivariable mixed-effects. At the bivariable analysis, variables with a p-value of less than 0.2 were included for the multivariable analysis. In the multivariable analysis variables with a *P*-value of less than 0.05 were considered as a factor associated with HIV testing among young women [5, 14–22].

## Results

### Socio-demographic characteristics of study participants

More than half (53.64%) of the women were in the age group of 15-19 years. Around 49.06 % of the participant had primary education and 8.22% had no formal education. The majority of the participants (56.66%) did not have a stigmatized attitude towards people living with HIV / AIDS. Regarding sexual behavior, the majority of the participants (83.12 %) had no risky sexual behavior and only 4.23 % had higher risky sexual behavior. Considering household wealth status, 45.36% were from the richest households. Approximately half (50.41%) of the respondents were from the community with a low level of education (Table 3).

**Table 3:** Sociodemographic characteristics of the respondents in eastern Africa (*N*=73, 661)

Variables		Frequency (%)
Age (years)	15-19	39,510 (53.64%)
	20-24	34,150 (46.36%)
Highest education level	No education	6057 (8.22%)
	Primary education	36139 (49.06%)
	Secondary education	28595 (38.82%)
	Higher education	2865 (3.89%)
Wealth index	Poor	26326 (35.74%)
	Middle	13919 (18.9%)
	Rich	33416 (45.36%)
Risky sexual behavior	No risk	61224 (83.12%)
	Some risk	9319 (12.65%)
	High risk	3118 (4.23%)
HIV knowledge	Low knowledge	12860 (17.46%)
	High knowledge	29949 (40.66%)
	Comprehensive knowledge	30852 (41.88%)
Marital status	Unmarried	52533 (71.32%)
	Married	21128 (28.68%)
Media exposure	No	20670 (28.06%)
	Yes	52990 (71.94%)
Working status	Not working	36455 (49.49%)
	Working	37206 (50.51%)
Number of sexual partner	One	66170 (89.83%)
	More than one	7491 (10.17%)
Stigma indicator	No stigma	41738 (56.66%)
	Low stigma	14107 (19.15%)
	Moderate stigma	14386 (19.53%)
	High stigma	3429 (4.65%)
Residence	Urban	21052 (28.58%)
	Rural	52609 (71.42%)
Age at sex	Before 20 years	37820 (51.34%)
	At 20 and after years	35840 (48.66%)
Visit health facility	No	34670 (47.07%)
	Yes	38991 (52.93%)
Community-level education	Low level of un education	36531 (49.59%)
	High level of un education	37130 (50.41%)

### Random effects model

As indicated in Table 4, the null model's ICC value, which was 0.4 suggests that around 4% of the overall HIV testing variance was due to cluster variability. Furthermore, the MOR value in the null model was 1.42 suggests a substantial clustering of HIV testing across clusters occurred. Besides, the PCV (0.36) in the final model (model III) suggested that 36% of the variation in HIV testing was explained by both individual and community levels factors. The final model (Model III) was the best fit since it had the lowest deviance.

**Table 4:** Random effect model and model fitness for the assessment of HIV testing among reproductive-age women in eastern Africa

Parameter	Null model	Model I	Model II	Model III
ICC	0.41	0.024	0.037	0.026
PCV	Reff	0.06	0.14	0.36
MOR	1.42	1.3	1.38	1.32
model comparison				
Log likelihood	-50675.43	-33893.26	-50431.36	-33842.81
Deviance	101350.86	67787.32	100862.72	67685.62

### Prevalence of HIV testing among young women

**Fig. 1:**
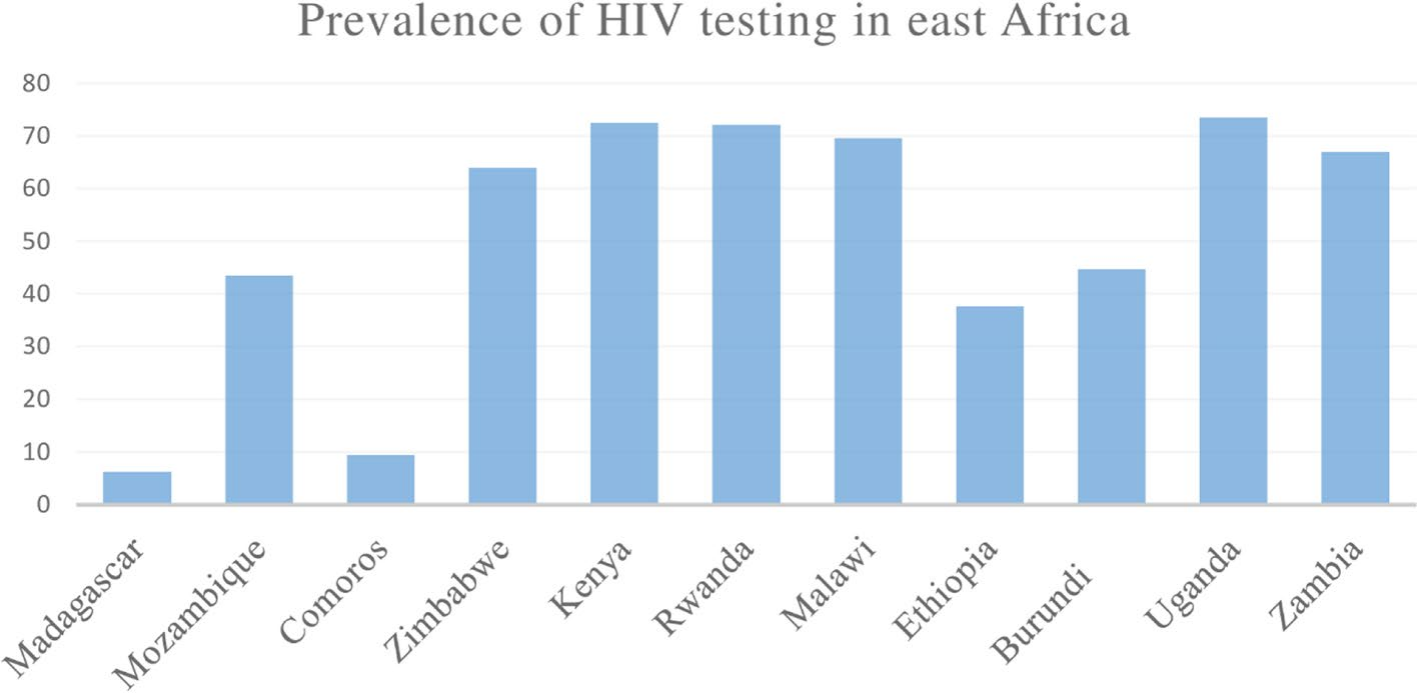
Showing prevalence of HIV testing among young women in east Africa

The prevalence of HIV testing and counseling among young women was 55.3 %( 95% CI = 54.97%, 55.69%) with the lowest prevalence (6.27%) in Madagascar and highest (73.51%) in Uganda (Fig 1).

### Factors associated with HIV testing among young women

We consider the final model for determining factors associated with HIV testing, as it had the lowest deviance. Women aged 20-24 years were more likely to be tested for HIV compared to women of early age. The chances of HIV testing were 2.48 (AOR= 2.49; 95 % CI=2.39, 2.58) times higher for women visiting health centers in the last 12 months compared with their counterparts. Looking at household wealth status, young women from rich households (AOR=0.86; 95 % CI=0.81, 0.91) had lower chances of being tested for HIV compared with young women from poor households. Married women were 1.88 times more likely to be tested for HIV compared to unmarried women (AOR=1.88; 95 % CI=1.79, 1.99). Individuals who initiate sex after 20 years of age had a 66 % lower odds of being tested for HIV compared to those who initiate sex at an earlier age (AOR=0.34; 95% CI=0.33, 0.36). Women from rural dwellers had 19% lower chances of being tested for HIV (AOR=0.80; 95% CI=0.77, 0.85). Regarding stigma attitude, there were 1.36 (AOR=1.36; 95% CI=1.24, 1.48), 1.63 (AOR=1.63; % CI=1.55, 1.71) and 1.59 (AOR=1.59; % CI=1.51, 1.67) times higher chances of being tested for HIV testing for young women with higher, moderate and low stigma score, respectively. Importantly, women with higher (AOR=5.6; 95% CI=5.27, 5.95) and comprehensive (AOR=8.04; 95% CI=7.55, 8.56) knowledge about HIV / AIDS were more likely to be tested for HIV compared to those with low knowledge. Women with a multiple-sex partner had 38% lower chances of being tested for HIV (AOR=0.62; 95% CI=0.52, 0.74) compared to their counterparts. Being from a community with a low educational level had 15 % lower chances of being tested for HIV (AOR=0.85; 95% CI=0.80, 0.90) (Table 5).

**Table 5:** Bivariable and multivariable multilevel binary logistic regression analysis of factors associated with HIV testing in east Africa in the final model

Variables		Ever tested for HIV		COR(95%CI)	AOR(95%CI)
		No	yes		
**Respondent age**	**15-19**	24127	15383	1	1
	**20-24**	8771	25380	4.58 (4.43, 4.73)	2.94 (2.81, 3.06)*
**Visiting health facility**	**No**	21581	13089	1	1
	**Yes**	11317	27674	3.93 (3.81, 4.06)	2.48 (2.39, 2.58)*
**Highest educational level**	**No education**	3767	2290	1	1
	**Primary education**	16874	19265	1.99 (1.88, 2.10)	2.40 (2.23, 2.59)*
	**Secondary education**	11596	17000	2.45 (2.32, 2.60)	3.03 (2.79, 3.28)*
	**Higher education**	662	2203	5.27 (4.76, 5.84)	3.59 (3.14, 4.10)*
**Wealth status**	**Poor**	12391	13935	1	1
	**Middle**	6447	7471	1.10 (1.05, 1.15)	0.94 (0.89, 1.00)
	**Rich**	14059	19357	1.21 (1.17, 1.25)	0.86 (0.81, 0.91)*
**Marital status**	**Unmarried**	27191	25342	1	1
	**Married**	5707	15421	2.96 (2.86, 3.07)	1.88 (1.79, 1.99)*
**Media exposure**	**No**	18062	9808	1	1
	**Yes**	22036	30955	1.54 (1.49, 1.59)	1.27 (1.21, 1.33)*
**No of sexual partner**	**One**	5131	4160	1	1
	**More than one**	27766	36603	1.55 (1.49, 1.62)	0.62 (0.52, 0.74)*
**HIV knowledge**	**Low knowledge**	4708	2550	1	1
	**Higher knowledge**	12849	17099	5.32 (5.07, 5.59)	5.6 (5.27, 5.95)*
	**Comprehensive knowledge**	9772	21080	8.25 (7.86, 8.67)	8.04 (7.55, 8.56)*
**Stigma indicator**	**Higher stigma**	1742	1687	1.00 (0.93, 1.08)	1.36 (1.24, 1.48)*
	**Moderate stigma**	5362	9024	1.75 (1.68, 1.83)	1.63 (1.55, 1.71)*
	**Low stigma**	5289	8818	1.79 (1.72, 1.87)	1.59 (1.51, 1.67)*
	**No stigma**	20504	21234	1	1
**Residence**	**Urban**	7703	13349	1	1
	**Rural**	25195	27714	0.73 (0.70, 0.75)	0.80 (0.77, 0.85)*
**Working statues**	**Not working**	18416	18064	1	1
	**Working**	12748	18218	1.50 (1.46, 1.55)	1.11 (1.07, 1.16)*
**Risky sexual behavior**	**Higher risk**	798	2319	2.83 (2.60, 3.07)	1.77 (1.55, 2.02)*
	**Some risk**	2876	6443	2.08 (1.98, 2.18)	1.20 (1.13, 1.27)*
	**No risk**	29223	32000	1	1
**Age at sex**	**Before 20 years**	10991	26830	1	1
	**At 20 and above years**	21907	13933	0.26 (0.25, 0.26)	0.34 (0.33, 0.36)*
**Community education**	**Low**	15083	21448	1	1
	**High**	17814	19316	0.69 (0.65, 0.73)	0.85 (0.80, 0.90)*

*p≤0.05

## Discussion

Most studies have shown that knowing one's serostatus helps to prevent and monitor the spread of infection [16]. The pooled prevalence of HIV testing among young women in east Africa was 55.3% (95% CI = 54.97%, 55.69%). The prevalence in this study was greater than the report in Ethiopia and sub-Saharan Africa [4, 8]. The prevalence in this study was smaller than many studies conducted in Africa [5, 11, 17–19]. Regional variations in access to HIV testing facilities as well as knowledge related to HIV / AIDS may also be the reasons for the reported regional inequalities in HIV testing implementation [18]. Young women who had primary and above education had a higher chance of being tested for HIV compared with women without formal education. This finding was supported by a study conducted elsewhere [4, 18, 20]. The explanation for this might be as education will enhance awareness about HIV and empowers women to make healthcare decisions by visiting the health facility [4]. Older women are more likely to get HIV tests than younger women. This is in line with the studies conducted in South Africa and sub-Saharan Africa [5, 8]. The variation in the odds of being tested for HIV among different age groups may be attributable to the possibility that younger women have a shorter sexual experience and are less knowledgeable about sexual issues than older people [10].

Married women had a higher chance of HIV testing than their counterparts, which is similar to the findings in Zambia and Ethiopia [4, 11]. This might be explained by the fact that married women used HIV counseling and testing better than unmarried women as they had regularly visited health facilities during pregnancy [12]. Young women living in rural areas had 20% lower odds of being tested for HIV compared to their urban counterparts. A similar conclusion was drawn from studies conducted in Ethiopia [4] and Nigeria [21]. Women attending health facilities were more likely to be tested for HIV compared to their counterparts. This finding was similar to studies conducted in South Africa [22, 23]. This may be because health professionals advise people who have visited health facilities to have HIV counseling and testing and this service is offered by almost all governmental and public health facilities [24]. Young women who had an experience of risky sexual activity were more likely to use HIV testing and counseling. The study conducted in Ethiopia and Ghana supported this finding [12, 25]. Besides, age at first sex is a significant predictor of HIV testing, which is in agreement with a study conducted in Malawi [17]. This may be explained as early age is associated with a higher risk of contracting various sexually transmitted diseases and risky sexual behavior, which could contribute to a higher risk of HIV infection [26]. Compared with those with one sexual partner, young women with multiple sexual partners had lower odds of being tested for HIV which is consistent with the study conducted in Ethiopia [4]. This might be because women with multiple sexual partners had a higher perceived risk of acquiring HIV infection which in turn, increases their motive to be tested [23]. Concerning HIV / AIDS knowledge, young women having higher and comprehensive knowledge about HIV had higher chances of being tested compared to women with low knowledge. This finding was supported by different studies [5, 8]. This might be justified by people with a good understanding of HIV / AIDS, including its transmission and preventive measures keeps themselves away from these devastating problems [13]. Looking at media exposure, there was a significant association between media exposure and HIV testing, which is supported by another study conducted in sub-Saharan Africa [8]. This outcome underlines the potential importance of media exposure for behavioral change, playing a key role in promoting young women's sexual and reproductive health [25].

### Strength and limitation of the study

The study has many strengths, first, the study was based on weighted nationally representative data from 11 east African countries with large sample size. Second, the analysis was using the multilevel analysis to accommodate the hierarchical nature of the DHS data to get reliable standard error and estimates. Moreover, since it is based on the national survey data the study has the potential to give insight for policy-makers and program planners to design appropriate intervention strategies both at national and regional levels. However, this study had limitations in that the DHS survey was based on respondents’ self-report, this might have the possibility of recall bias. Besides, since this study was based on cross-sectional DHS data, we are unable to show the temporal relationship between HIV testing and the independent variables.

## Conclusion

The pooled prevalence of HIV testing among young women is higher in east Africa compared to the report from other studies. In the multilevel multivariable analysis; respondent age, marital status, educational level, knowledge about HIV/AIDS, HIV stigma indicator, risky sexual activity, women who have visited health care facilities, early sex initiation and community educational level were positively associated with HIV testing among young women. While being a rural dweller, wealth status and being having multiple sexual partners were negatively associated with HIV testing and counseling. So there should be a systematic and integrated approach and policy for HIV counseling and testing among young women to prevent the transmission and spread of HIV/AIDS in these populations. Besides, all the stakeholders should have an integrated approach to increase awareness of the benefit of HIV testing and counseling to control the spread of HIV/AIDS.

## Data Availability

The data set is available online and anyone can access it from www.measuredhs.com.

## Abbreviations

CI: Confidence Interval
CSA: Central Statistical Agency
DHS: Demographic Health Survey
EA: Enumeration Area
ICC: Interclass correlation
LLR: Likelihood Ratio
STI: Sexually transmitted infection
WHO: World Health Organization
